# Household costs and time to treatment for children with severe febrile illness in rural Burkina Faso: the role of rectal artesunate

**DOI:** 10.1186/s12936-018-2526-8

**Published:** 2018-10-22

**Authors:** Joëlle Castellani, Borislava Mihaylova, Mohamadou Siribié, Zakaria Gansane, Amidou Z. Ouedraogo, Florence Fouque, Sodiomon B. Sirima, Silvia M. A. A. Evers, Aggie T. G. Paulus, Melba Gomes

**Affiliations:** 1 Department of Health Services Research, Care and Public Health Research Institute (CAPHRI), Maastricht University, Maastricht, The Netherlands; 2 Health Economics Research Centre, Nuffield Department of Population Health, University of Oxford, Oxford, UK; 3 Centre for Primary Care and Public Health, Barts and the London School of Medicine and Dentistry, Queen Mary University of London, London, UK; 4 Groupe de Recherche Action en Santé (GRAS), Ouagadougou, Burkina Faso; 5 UNICEF/UNDP/World Bank/WHO Special Programme for Research & Training in Tropical Diseases (TDR), World Health Organization, Geneva, Switzerland

**Keywords:** Malaria, CHW, Rectal artesunate, RDTs, Economics, Costs and cost analysis, Time to treatment

## Abstract

**Background:**

Community health workers (CHWs) were trained to identify children with malaria who could not take oral medication, treat them with rectal artesunate (RA) and refer them to the closest healthcare facility to complete management. However, many children with such symptoms did not seek CHWs’ care. The hypothesis was that the cost of referral to a health facility was a deterrent. The goal of this study was to compare the out-of-pocket costs and time to seek treatment for children who sought CHW care (and received RA) versus those who did not.

**Methods:**

Children with symptoms of severe malaria receiving RA at CHWs and children with comparable disease symptoms who did not go to a CHW were identified and their parents were interviewed. Household out-of-pocket costs per illness episode and speed of treatment were evaluated and compared between RA-treated children vs. non-RA treated children and by central nervous symptoms (CNS: repeated convulsions, altered consciousness or coma).

**Results:**

Among children with CNS symptoms, costs of RA-treated children were similar to those of non-RA treated children ($5.83 vs. $4.65; p = 0.52), despite higher transport costs ($2.74 vs. $0.91; p < 0.0001). However, among children without CNS symptoms, costs of RA-treated children were higher than the costs of non-RA treated children with similar symptoms ($5.62 vs. $2.59; p = 0.0001), and the main driver of the cost difference was transport. After illness onset, CNS children reached CHWs for RA an average of 9.0 h vs. 16.1 h for non-RA treated children reaching first treatment [difference 7.1 h (95% CI − 1.8 to 16.1), p = 0.11]. For non-CNS patients the average time to CHW-delivered RA treatment was 12.2 h vs. 20.1 h for those reaching first treatment [difference 7.9 h (95% CI 0.2–15.6), p = 0.04]. More non-RA treated children developed CNS symptoms before arrival at the health centre but the difference was not statistically significant (6% vs. 4%; p = 0.58).

**Conclusions:**

Community health worker-delivered RA does not affect the total out-of-pocket costs when used in children with CNS symptoms, but is associated with higher total out-of-pocket costs when used in children with less severe symptoms. RA-treated children sought treatment more quickly.

**Electronic supplementary material:**

The online version of this article (10.1186/s12936-018-2526-8) contains supplementary material, which is available to authorized users.

## Background

Children with severe disease deteriorate rapidly and die. Malaria deaths can be prevented by prompt treatment. Rectal artesunate (RA) is effective in preventing malaria deaths when given to children with danger signs (defined as inability to eat, drink or breastfeed, repeated vomiting, lethargy, convulsions, altered consciousness or coma) [1, 2] together with a referral to the nearest hospital to complete management [3].

The World Health Organization’s strategy for child survival, the Integrated Management of Childhood Illness (WHO-IMCI), focuses on the major childhood causes of mortality, including malaria [2, 4, 5]. In the community component of this strategy (often referred to as integrated community case management (iCCM) [6]), several interventions have been implemented to improve access to health care for children living in poor and remote areas [7]. In malaria endemic areas, under iCCM, community health workers (CHWs) are trained to recognize children with danger signs, to diagnose, and, if malaria positive, to treat with RA and refer the child to the closest healthcare facility for further management [5, 8].

Studies have investigated why children with uncomplicated fever, malaria or childhood illness in rural settings do not seek care at a CHW [9–14]. Often the symptoms were not perceived as severe and warranting CHW intervention [12, 14], drugs were out-of-stock [11–13], or parents had more confidence in the health centres and their drug supplies, personnel and training [11, 13, 14]. Research examined fatal episodes and utilization of CHWs/modern biomedical care during severe fatal illness in Ethiopia, Uganda and Tanzania [15–17]. In the Ethiopian study [15], delays in seeking care were attributed to the waxing and waning of children’s symptoms which delayed seeking care, and inadequate knowledge about the need for early diagnosis and treatment whereas in the Tanzanian study the majority (78.7%) sought modern biomedical care, often from more than one provider; CHWs were not mentioned [17]. In both Uganda and Tanzania caregivers sought facility care [16, 17] with a minority using traditional care [17]. Other studies have reported that the majority of children who either die from severe illness or whose illness was recognized as being severe by the caregiver did not receive adequate treatment [18, 19], with one of the studies attributing this partially to the caregivers’ inability to pay for the costs of transport and treatment at a facility [19]. Household and health system costs of malaria episodes (uncomplicated or severe) have been estimated and modelled for 3 African countries showing that malaria household costs increase with disease severity and hospitalization [20]. Unfortunately, as pointed out in a 2003 review of the economic costs of malaria [21], direct information on household costs of severe malaria is lacking, partly because the condition is rare, making it difficult to obtain data through representative household surveys in a community, and because its symptoms (e.g. convulsions, loss of consciousness) may not lead to the condition being reported as malaria.

For children with severe illness, understanding the speed of treatment and referral is essential as delays in seeking care for severe malaria are associated with higher risk of death. Such a study also provides an important indicator of the quality of malaria interventions where the goal is to achieve malaria treatment within 24 h. However little is known about the speed of seeking treatment at CHWs or compliance with referral advice. One study reported that 1/3 of children who were urgently referred by drug distributors experienced delays of more than 24 h [22].

Illness episodes impose costs on carers of sick children and these anticipated costs might influence their health-seeking behaviour. While several studies have assessed the out-of-pocket costs of an episode of severe illness [23–26], there is no information on the out-of-pocket costs of households when RA is used and whether these costs might act as a deterrent. One study evaluated the cost-effectiveness of RA [27] and another assessed the out-of-pocket and societal costs for children who completed referral in an area where CHW-delivered RA was used [28]. A further study reported that children who received RA had poor adherence to referral to fee-based facilities [29]. The goal of this study was thus to evaluate household out-of-pocket costs for an episode of severe illness in children who received CHW-delivered RA compared to similar illness episodes in children who did not seek CHW’s care.

## Methods

### Study area and population

The study was undertaken in Burkina Faso and was nested within an intervention implementing malaria rapid diagnosis tests (RDTs) and oral and RA malaria treatment in malaria endemic villages via CHWs in the rural area of Sidéradougou, Mangodara District in 2015 [30].

For this study, children who received CHW-delivered RA-treatment and children who did not were identified, and the out-of-pocket costs incurred by their families, clinical outcomes (i.e. deterioration to CNS symptoms by arrival at health centre) and the time from illness onset to treatment were compared. By definition, children who did not receive RA did not attend a CHW for diagnosis and treatment. Comparisons were made between children with similar illness severity (such as repeated vomiting, lethargy, convulsions or altered consciousness/coma), but there was no attempt to sample similar number of children in different categories.

Guardians of children 6–59 months of age in the intervention villages with a fever in the past 2 weeks were randomly selected for interview without knowledge of whether the children had uncomplicated or severe illness or sought CHW care [30]. Households without sick children, whose caregiver was not present during the illness or refused consent were excluded from interview.

**Fig. 1:**
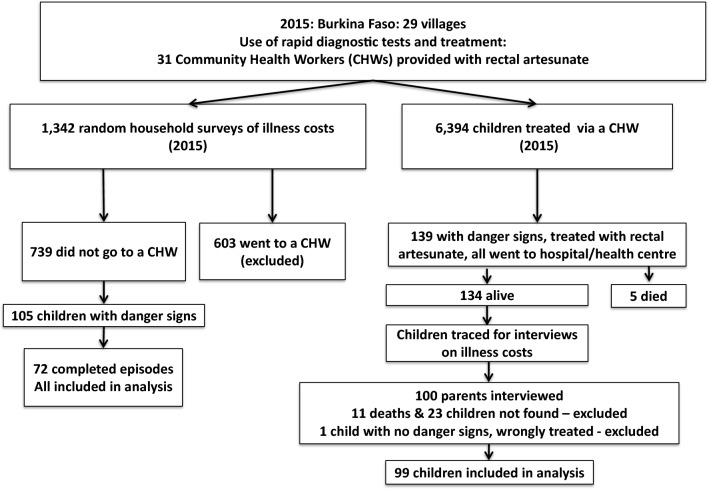
Identifying children with danger symptoms who received or not rectal artesunate

Children with similar symptoms to RA-treated children, who did not seek CHW help, were identified from these random household sampling surveys and only illness episodes which had completed at the time when the parent or carer was questioned about out-of-pocket costs were included in the analysis. 1342 household interviews were conducted [30], but only 72 severe episodes contributed to this analysis by meeting the eligibility criteria of a completed illness episode in a child with danger signs warranting RA treatment, but where RA treatment was not sought (Fig. 1). Households were asked questions regarding the illness symptoms, treatment-seeking behaviour (healthcare providers visited, date and clock time visited, treatments provided) and the associated out-of-pocket costs.

Identical data on illness, treatment-seeking behaviour and costs was obtained from RA-treated children 6–59 months, who were identified from the CHWs’ treatment record forms. A total of 139 children received RA in Burkina Faso, 134 of whom were alive at the end of the episode (Fig. 1) [30]. At the time of study interviews (mid-2016), a further 11 children had died (and were excluded from the study as it was considered culturally insensitive to seek out-of-pocket cost data after a death); 23 children had moved residence and were lost to follow up; and one child was inappropriately treated with RA. Thus, study data on a total of 99 RA-treated children with danger signs during illness episode was available.

### Healthcare provision in the study area

In Burkina Faso, the “*Centre de santé et de promotion sociale* (CSPS)” is the first level of care for patients. CSPS are managed by nurses in outpatient facilities. Patients needing specialized care are referred to a “*centre médical*” (health centre) where admission and supervision by medical doctors is available; referral to the regional hospital occurs if required. There is only one health centre in the study area. However, there are many traditional healers, CHWs, dispensaries/CSPS, and drug hawkers in addition to shops and pharmacies which sell quinine, antibiotics and anti-malarials (excluding the artemisinin derivatives) without prescription.

At the time of the study, health care provided at public facilities was fee-based. The cost of consultation was about 200 West African CFA francs (XOF; US Dollars [USD] $0.33) and when admitted, the patient had to pay for their bed; no other costs were charged to the patient.

### CHW training

As part of the intervention, CHWs were trained to diagnose malaria using RDTs at no cost to the patient, recognize severe illness (where children were unable to take oral medication and had “danger signs” requiring immediate attention—inability to eat, drink or suck; repeated vomiting; lethargy; convulsions or altered consciousness/coma) and treat malaria positive patients. For children with danger signs, free RA was provided, and all RA-treated patients were referred to the closest healthcare facility for further management, with an explanation of the importance of reaching the facility quickly, to complete patient management.

Community health worker training was in accordance with WHO Guidelines on IMCI which require that children with fever or a history of fever within the previous 24 h are assessed by a CHW. Training lasted 3 days and was evaluated through pre- and post-tests [31]. 31 CHWs passed certification for delivery of RA in 29 rural villages [30].

### Questionnaires and data

Case report forms (CRFs) were developed in French and pilot tested before use. In CRFs, information was sought about the characteristics of the child and households (sex, age, education, occupation, household food scarcity, the number of working people over 10 years old), the date and clock time from onset of each symptom, the date and clock time of visit (for each provider visited) and the out-of-pocket costs associated with each visit. Guardians provided detail about the costs incurred at each healthcare provider for registration, consultation, diagnosis, drugs, bed, food/drinks and transport [14, 24–26]. All data were reported by the guardians from memory and the same interviewers were used for all household interviews to minimize interviewers’ bias. Identical information on illness, treatment-seeking behaviour and household costs was obtained for RA-treated and non-RA treated patients. However, interviews for RA-treated patients did not occur within 2 weeks of the episode, but at the end of the intervention period.

### Data analysis

For both RA and non-RA treated children, their illness symptoms were classified into cerebral symptoms involving the central nervous system (CNS—convulsions, coma or altered consciousness) and non-CNS symptoms (lethargy and/or repeated vomiting). Baseline characteristics, the out-of-pocket costs and the duration of time from onset of symptoms to seeking treatment were compared between RA and non-RA treated children categorized by CNS status.

#### Out-of-pocket costs

Only direct out-of-pocket costs from onset of illness to recovery were collected and analysed. The opportunity cost of time taken by guardians to look after the child was beyond the scope of the study. In a sensitivity analysis, the costs of non-RA treated patients who attended a health centre were also analysed. All data on costs were collected in XOF and were converted and presented in USD using the average exchange rate between April and October 2015: 1 USD = 598.09 XOF (http://www.oanda.com).

#### Time delays in seeking treatment

Because children with severe malaria deteriorate fast and die, time delays were calculated from clock times and dates provided by carers on the clinical course of illness and actions taken. From the clock times and dates reported by the main caregiver of the child, the duration of delay (in hours) was calculated: from onset of illness to arrival at 1st modern healthcare provider outside the home (CHWs, drug shops, dispensaries, health centres) and from onset of illness (and onset of danger signs) to arrival at the health centre. The time from administration of RA to arrival at health centre was calculated.

#### Deterioration

For both groups, deterioration was defined as the onset of CNS symptoms [32] prior to arrival at the health centre after a visit to a healthcare provider. For children in the RA-treatment group, this provider was the CHW.

### Statistical methods

Data were double entered in EpiData 3.1 and analysed using Stata software, version 13.0 (StataCorp, College Station, Texas). A Student t-test was performed on the equality of means and a test of proportion on the equality of proportion with a level of significance of p = 0.05 and a confidence level of 95%, and either a test for heterogeneity or a linear trend of odds (if there were more than two ordered groups) was used to compare baseline characteristics of those who received RA vs. those who did not, by CNS status.

## Results

### Baseline characteristics

Table 1 presents the baseline characteristics of the children, caregivers and illness episodes for RA-treated children and non-RA treated children by CNS status. Most children in both RA and non-RA groups were less than 36 months of age (86.9% RA; 59.7% non-RA). More than 75% of the guardians had no education and for the remaining, although not statistically significant, the guardians of RA-treated children were more educated than the guardians of non-RA treated children (e.g.: 2.0% RA vs. 0.0% non-RA treated guardians reported having > 7 years of education).

**Table 1:** Baseline characteristics, by symptoms: children treated with RA versus children not treated with RA

Category	CNS + other symptoms^a^				No CNS but prostrated with other symptoms^a^				Total			
	RA		No RA		RA		No RA		RA		No RA	
	N	%	N	%	N	%	N	%	N	%	N	%
Total no. of children	30	–	24	–	69	–	48	–	99	–	72	–
Child characteristics												
Child’s age, months												
< 36 mo	25*	83.3	13	54.2	61*	88.4	30	64.6	86*	86.9	43	59.7
≥ 36 mo	5*	16.7	11	45.8	8*	11.6	18	35.4	13*	13.1	29	40.3
Child’s gender												
Male	14	46.7	13	54.2	32	46.4	27	56.3	46	46.5	40	55.6
Female	16	53.3	11	45.8	37	53.6	20	41.7	53	53.5	31	43.1
Missing	–	–	–	–	–	–	1	2.1	–	–	1	1.4
Caregiver and household characteristics												
Caregiver’s gender												
Male	3	10.0	–	–	24*	34.8	–	–	27*	27.3	–	–
Female	27	90.0	24	100.0	45*	65.2	48	100.0	72*	72.7	72	100.0
Caregiver’s age, years												
15–24	1*	3.3	6	25.0	11*	15.9	16	33.3	12*	12.1	22	30.6
25–35	15*	50.0	14	58.4	37*	53.6	26	54.2	52*	52.5	40	55.6
36–50	11*	36.7	2	8.3	19*	27.6	3	6.2	30*	30.3	5	6.9
> 50	–	–	–	–	2*	2.9	–	–	2*	2.0	–	–
Missing/unknown	3	10.0	2	8.3	–	–	3	6.2	3	3.0	5	6.9
Education												
No education	23	76.7	23	95.8	56	81.2	40	83.3	79	79.8	63	87.5
≤ 7 years	7	23.3	1	4.2	9	13.0	8	16.7	16	16.2	9	12.5
> 7 years	–	–	–	–	2	2.9	–	–	2	2.0	–	–
Missing/unknown	–	–	–	–	2	2.9	–	–	2	2.0	–	–
Occupation												
Unemployed	–	–	–	–	–	–	3	6.2	–	–	3	4.2
Agriculture	30*	100.0	16	66.7	68*	98.6	25	52.1	98*	99.0	41	56.9
Self-employed (only or + agriculture)	–	–	8	33.3	–	–	20	41.7	–	–	28	38.9
Missing/unknown	–	–	–	–	1*	1.4	–	–	1*	1.0	–	–
Household food scarcity												
Never	22	73.3	18	75.0	48*	69.6	32	66.7	70*	70.7	50	69.5
Seldom	–	–	4	16.7	–	–	10	20.8	–	–	14	19.4
Sometimes	1	3.3	2	8.3	1*	1.4	6	12.5	2*	2.0	8	11.1
Often	–	–	–	–	2*	2.9	–	–	2*	2.0	–	–
Always	6	20.0	–	–	16*	23.2	–	–	22*	22.2	–	–
Missing/unknown	1	3.3	–	–	2*	2.9	–	–	3	3.0	–	–
No. of working people over 10 years of age in the household												
≤ 2	10	33.3	13	54.2	20*	29.0	24	50.0	30*	30.3	37	51.4
3–5	13	43.3	10	41.7	35*	50.7	23	47.9	48*	48.5	33	45.8
6–10	5	16.7	1	4.1	9*	13.0	1	2.1	14*	14.1	2	2.8
> 10	1	3.3	–	–	2*	2.9	–	–	3*	3.0	–	–
Missing/unknown	1	3.3	–	–	3*	4.4	–	–	4	4.0	–	–
Illness characteristics												
Providers visited^b^												
Traditional medicine only	–	–	3^c^	12.5	–	–	10^c^	20.8	–	–	13^c^	18.1
Itinerant drug sellers/home stock only	–	–	7	29.2	–	–	17	35.4	–	–	24	33.3
Traditional medicine and home stock	–	–	1	4.2	–	–	–	–	–	–	1	1.4
1 “modern” provider^d^	–	–	12	50.0	–	–	20	41.7	–	–	32	44.5
2 “modern” providers^d^	29	96.7	1	4.2	69	100.0	–	–	98	99.0	1	1.4
3 “modern” providers^d^	1	3.3	–	–	–	–	–	–	1	1.0	–	–
No. days lost caring for sick children: completed episode												
≤ 2 d	12	40.0	8	33.3	8*	11.6	20	41.7	20*	20.2	28	38.9
> 2–5 d	14	46.7	15	62.5	51*	73.9	26	54.2	65*	65.7	41	56.9
> 5–8 d	4	13.3	1	4.2	9*	13.0	2	4.1	13*	13.1	3	4.2
> 8 d	–	–	–	–	1*	1.5	–	–	1*	1.0	–	–

*RA* rectal artesunate, *CNS* central nervous system (convulsions; altered consciousness/coma), *mo* months, *d* days

* Test of heterogeneity: RA vs. no RA: p < 0.05

^a^Repeated vomiting ± too weak to take oral medication/“lethargy”

^b^1 child without CNS did not go to any provider

^c^CNS: 1 child went to a traditional healer and 2 used local herbs at home; No CNS: 2 children went to a traditional healer, 8 used local herbs at home; Total: 3 children went to a traditional healer; 10 used local herbs at home

^d^“Modern” defined to include community health workers, drug shops, dispensaries, health centres, and hospitals for RA children and drug shops, dispensaries, and health centres for non-RA children. Traditional or informal care providers could have been used before modern providers

Most households’ income was derived from agriculture (99.0% RA; 56.9% non-RA) but in 38.9% of the households of non-RA treated children, income was based on self-employment. RA-treated children went to a larger number of “modern” providers (> 1 provider: 100.0% RA vs. 1.4% non-RA), lived in households with higher number of working people (> 5 working people: 17.1% RA vs. 2.8% non-RA), but lacked more food than non-RA families (household food scarcity: often or always: 24.2% RA vs. 0.0% non-RA). Finally, most of the guardians (in both groups) spent less than 5 days in caring for their sick child (85.9% RA; 95.8% non-RA).

### Household out-of-pocket costs for illness episode

All RA-treated patients incurred some costs during the illness episode (see Additional file 1). In the non-RA treated group, 83% of the patients with CNS and 77% without CNS incurred some costs, but in the case of non-RA treated children who went to a health centre, they all incurred costs mainly because they paid for transport to the facility (87%), and the remaining 13% who did not incur transport costs paid for medications.

Table 2 presents the household costs by cost category, for those who sought CHW care and were treated with RA and those who did not, by CNS status. Among patients who were not treated with RA, costs are presented also separately for those who went to a health centre. All patients treated with RA delivered by a CHW were referred to the health centre and all of them completed subsequent referral.

**Table 2:** Mean out-of-pocket costs (US Dollars) for completed episodes of severe illness, by symptoms: children treated with RA versus children not treated with RA

Category	CNS + other symptoms^a^						No CNS but prostrated with other symptoms^a^						Total					
	RA		No RA		No RA but went to a health centre		RA		No RA		No RA but went to a health centre		RA		No RA		No RA but went to a health centre	
	N	Mean (SD)	N	Mean (SD)	N	Mean (SD)	N	Mean (SD)	N	Mean (SD)	N	Mean (SD)	N	Mean (SD)	N	Mean (SD)	N	Mean (SD)
Costs in USD																		
Registration	30	–	24	–	11	–	69	0.005 (0.04)	48	–	20	–	99	0.003 (0.03)	72	–	31	–
Consultation	30	–	24	0.15 (0.17)	11	0.32 (0.05)	69	–	48	0.09 (0.15)	20	0.21 (0.16)	99	–	72	0.11 (0.16)	31	0.25 (0.14)
Diagnosis	30	0.34 (1.86)	24	–	11	–	69	–	48	–	20	–	99	0.10 (1.02)	72	–	31	–
Drugs before health centre	30	0.05 (0.08)	24	0.75 (2.17)	11	0.03 (0.07)	69	0.03 (0.06)	48	0.20 (0.57)*	20	0.01 (0.04)	99	0.03 (0.07)	72	0.38 (1.35)*	31	0.02 (0.05)
Drugs at the health centre^b^	30	1.02 (3.76)	11	4.93 (3.01)*	11	4.93 (3.01)*	69	1.35 (3.01)	20	4.04 (4.12)*	20	4.04 (4.12)*	99	1.25 (3.24)	31	4.36 (3.74)**	31	4.36 (3.74)**
Bed	30	0.11 (0.62)	24	0.12 (0.33)	11	0.26 (0.46)	69	0.05 (0.39)	48	–	20	–	99	0.07 (0.47)	72	0.04 (0.20)	31	0.09 (0.29)
Food^c^	30	1.57 (1.81)	24	0.47 (1.03)*	11	0.97 (1.38)	69	1.82 (2.43)	48	0.05 (0.18)**	20	0.12 (0.26)*	99	1.74 (2.26)	72	0.19 (0.64)**	31	0.42 (0.92)*
Other	30	–	24	–	11	–	69	–	48	0.01 (0.07)	20	0.03 (0.11)	99	–	72	0.01 (0.06)	31	0.02 (0.09)
Transport	30	2.74 (1.05)	24	0.91 (1.08)**	11	1.57 (1.03)*	69	2.38 (1.00)	48	0.56 (0.77)**	20	1.31 (0.67)**	99	2.49 (1.03)	72	0.68 (0.89)**	31	1.40 (0.81)**
Total costs	30	5.83 (7.82)	24	4.65 (4.79)	11	8.09 (3.96)	69	5.62 (4.18)	48	2.59 (3.90)**	20	5.72 (4.37)	99	5.68 (5.50)	72	3.27 (4.29)*	31	6.56 (4.32)

*RA* rectal artesunate, *CNS* central nervous system (convulsions; altered consciousness/coma), *SD* standard deviation, *USD* US Dollars

* Reference group: RA—p < 0.05

** Reference group: RA—p < 0.001

^a^Repeated vomiting ± too weak to take oral medication/“lethargy”

^b^For the non-RA group, only those who went to a health centre were included. Therefore, the results are the same as for the non-RA group who went to a health centre

^c^Food for the patient and accompanying family members

#### Total costs

For CNS patients, total costs were similar for RA-treated and non-RA treated patients, regardless of whether the latter went to a health centre ($5.83 vs. $4.65 p = 0.52) (Table 2). For non-CNS patients, RA-treated children incurred significantly higher total costs than non-RA treated children and the main driver of the cost difference was transport to a health centre and/or to a CHW: Total costs for non-CNS children: $5.62 for RA-treated vs. $2.59 for non-RA treated [difference $3.03 (95% CI 1.51–4.54), p = 0.0001]. For patients who went to a facility, there was no significant difference in costs between RA-treated and non-RA treated patients.

#### Transport costs

Children treated with RA had significantly higher mean transport costs than children who were not because all RA-treated children completed referral at the health centre (Table 2). Transport was between 3 and 4.25 times higher for the RA group compared with all patients in the non-RA group, but only 1.7 times higher compared with the non-RA children who went to a facility. For CNS children the average transport cost was $2.74 for RA-treated patients vs. $0.91 for non-RA treated patients [difference $1.83 (95% CI 1.25–2.42); p < 0.0001] and for non-CNS children, the transport cost was $2.38 for RA-treated patients vs. $0.56 for non-RA treated patients [difference $1.82 (95% CI 1.48–2.16), p < 0.0001]. For CNS patients who went to a facility the transport cost difference was just over one US dollar between the 2 groups: $2.74 for RA-treated patients vs. $1.57 for non-RA treated patients [difference $1.17 (95% CI 0.42–1.91), p = 0.0031]. For non-CNS patients who went to a facility the difference was similar: $2.38 vs. $1.31 or $1.07 difference [(95% CI 0.59–1.54), p < 0.0001].

#### Drug costs

It is important to note that non-RA treated patients who went to the single referral health centre in the District had higher mean drug costs at the facility than RA patients (Table 2). In general, drug costs at the facility constituted around 66% of the total episode costs for non-RA patients and 22% for the RA group. For CNS patients who went to a health centre the drug cost difference was $3.91: $1.02 vs. $4.93 [(95% CI 1.36–6.47), p = 0.0036]. For non-CNS patients the difference was lower, but still significant $2.69: $1.35 vs. $4.04 [(95% CI 1.04–4.36), p = 0.0017].

#### Other costs

For the other categories (registration, consultation, diagnosis, bed), costs were minor for both the RA and non-RA groups while food costs were in general nine times higher for the RA group vs. the non-RA group: $1.74 vs. $0.19 [difference $1.55 (95% CI 1.01–2.09), p < 0.0001] (Table 2).

### Speed of treatment

Table 3 reports the time taken in hours to seek treatment for those who were treated with RA vs. those who were not, by CNS status. Almost half of the non-RA treated children went to a “modern” healthcare provider and were taken there within 24 h. However, comparing the mean time to obtain treatment from illness onset it is clear that RA-treated patients sought treatment substantially faster, usually in less than half the time of those not treated with RA. For CNS RA-treated children, the average time to RA treatment was 9.0 h vs. 16.1 h for those treated with other medications [time difference 7.1 h (95% CI − 1.8 to 16.1), p = 0.11] and for non-CNS patients, the average time was 12.2 h for RA-treated children vs. 20.1 h for non-RA treated children [time difference 7.9 h (95% CI 0.2–15.6), p = 0.04]. For RA-treated children, the average referral time to reach the health centre after RA was 3.2 h.

**Table 3:** Time taken in hours to seek treatment and deterioration of clinical status, by symptoms: children treated with RA versus children not treated with RA

Category	CNS + other symptoms^a^				No CNS but prostrated with other symptoms^a^				Total			
	RA		No RA		RA		No RA		RA		No RA	
	N	% or mean (SD)	N	% or mean (SD)	N	% or mean (SD)	N	% or mean (SD)	N	% or mean (SD)	N	% or mean (SD)
“Modern” provider^b^												
Yes	30	100%	13^c^	54%	69	100%	20	42%	99	100%	33^c^	46%
No	–	–	11	46%	–	–	28	58%	–	–	39	54%
From onset of illness to 1st “modern” provider: Was the child treated within 24 h?												
Yes	26	87%	9	69%	59	86%	14	70%	85	86%	23	70%
No	4	13%	4	31%	10	14%	6	30%	14	14%	10	30%
Mean time (h)^d, e^	30	9.0 (9.9)	9	16.1 (16.5)	69	12.2 (12.0)*	14	20.1 (18.3)	99	11.2 (11.5)*	23	18.5 (17.3)
Deterioration: CNS on arrival^f^	4/30	13%	2/11	18%	–	–	–	–	4/99	4%	2/31	6%
From onset of illness to health centre: mean time (h) to reach a health centre^g^	30	14.5 (10.7)	7	17.2 (17.7)	68	15.2 (12.0)	14	20.1 (18.3)^h^	98	15.0 (11.5)	21	19.1 (17.7)
From danger signs to health centre^i^ : mean time (h) to reach a health centre^j, k^	22	10.4 (10.3)	7	12.0 (18.7)	54	11.3 (11.5)	12	14.8 (18.6)	76	11.1 (11.1)^k^	19	13.7 (18.2)

*RA* rectal artesunate, *CNS* central nervous system (convulsions; altered consciousness/coma), *SD* standard deviation, *h* hours

* RA vs. no RA: p < 0.05

^a^Repeated vomiting ± too weak to take oral medication/“lethargy”

^b^“Modern” defined to include community health workers, drug shops, dispensaries, health centres, and hospitals for RA children and drug shops, dispensaries, and health centres for non-RA children. Traditional or informal care providers were excluded

^c^CNS: 11 children went to a health centre, 1 to a drug shop and 1 to a dispensary/CSPS; Total: 31 children went to a health centre, 1 to a drug shop and 1 to a dispensary/CSPS

^d^CNS: missing for 4 children with no RA; No CNS: missing for 6 children with no RA; Total: missing for 10 children with no RA

^e^Mean time from onset of illness to 1st provider (home stock, traditional healer, drug hawkers) for children who did not go to a “modern” provider: CNS and no RA: N = 8, Mean = 2.0, SD = 3.1, missing for 3 children; No CNS and no RA: N = 17, Mean = 12.3, SD = 11.5, missing for 10 children, 1 child did not go to any provider; Total and no RA: N = 25, Mean = 9.0, SD = 10.7, missing for 13 children, 1 child did not go to any provider

^f^Symptoms after baseline, documented at health centre

^g^CNS: missing for 4 children with no RA; No CNS: missing for 1 child with RA and 6 children with no RA; Total: missing for 1 child with RA and 10 children with no RA

^h^Same as the mean time from onset of illness to 1^st^ “modern” provider because the health centre was the only “modern” provider visited

^i^Any danger signs (coma/altered consciousness, convulsions, repeated vomiting, lethargy) for the CNS and total groups

^j^CNS: missing for 8 children with RA, 4 children with no RA; No CNS: missing for 15 children with RA, 8 children with no RA; Total: missing for 23 children with RA, 12 children with no RA

^k^Mean referral time (h) from RA to the health centre: Total—RA: 3.2 h

In general, although not significant in this study, non-RA treated patients took more time to reach a health centre after onset of illness: 15.0 h vs. 19.1 h [time difference 4.1 h (95% CI − 2.0–10.2), p = 0.18], and a higher through not statistically significant different percentage of them deteriorated to one or more CNS symptoms by arrival (6% vs. 4%; p = 0.58).

## Discussion

This study examines the out-of-pocket costs and time to seek treatment for children with severe febrile illness. Children treated with CHW-delivered RA were compared with children who were not treated with RA in categories by cerebral malaria (CNS) status. Parents of children with CNS symptoms of repeated convulsions, altered consciousness or coma, treated with RA did not pay more than non-RA treated children. There was a trend for RA-treated children with CNS to reach the only healthcare facility more quickly, and consequently higher transport was their main expenditure. Despite higher transport costs, they had equivalent total costs to the CNS non-RA group, primarily because the latter spent significantly more on drugs. The higher costs may have been attributable to clinical deterioration, but this could not be confirmed.

The lack of a difference in out-of-pocket costs for RA-treated and not treated children with CNS symptoms is an important finding. Firstly, children with CNS symptoms are at very high risk of death [33, 34]. Secondly, the requirement to proceed to hospital/health centre after treatment is essential, but often perceived to be a major cost deterrent to compliance with referral advice, thus potentially delaying assessment and definitive treatment at the referral facility [35, 36]. In this study, trained CHWs explained the importance of referral and emphasized the need for rapid compliance with referral advice. Their consistent advice was heeded, even though parents probably spent more for transport in order to arrive rapidly at the referral facility [37] as emergency transfer usually costs more than non-emergency transport [38]. Among patients with CNS symptoms, the transport and food costs of RA-treated children were three times higher than those of non-RA treated patients. However, parents of non-RA treated children arrived around 3 h later at the health centre, and paid almost five times more on drugs at the health centre than parents of RA-treated patients.

In contrast, although not significant in this study, children with CNS symptoms who did not seek CHW-delivered RA but eventually went to a health centre not only took a longer time from the first symptom to consult a “modern” provider (9.0 h vs. 16.1 h) but also 1.19 times longer than RA-treated children with CNS symptoms to reach a health centre (14.5 h vs. 17.2 h). A higher, not statistically significant, proportion of children (6%) not treated with RA deteriorated to CNS symptoms by arrival at the health centre compared to RA-treated patients (4%). Since children were seen at the same health centre, it is possible that the clinical deterioration of the non-RA treated patients contributed to their higher drug expenditures at the centre compared with RA-treated children with CNS symptoms ($1.02 vs. $4.93).

The cost pattern and time delays for children without CNS symptoms (e.g. children with repeated vomiting/lethargy but at lower risk of death [32, 33]) presented a different picture. Time delays from onset of illness to the first “modern” healthcare provider were significantly longer (12.2 h vs. 20.1 h) and episode costs were significantly lower for non-RA treated children than for RA-treated children without CNS symptoms, probably because low-cost traditional treatments or home treatments were used [39]. RA-treated children incurred twice higher costs, mainly because of transport costs to a facility. However, it was noted that non-RA treated patients spent substantially more on drugs at the health centre, possibly because of the deteriorating condition of the child at arrival.

The ideal is that all parents of children with danger signs recognize the gravity of the symptoms and proceed to a CHW or health centre as quickly as possible for diagnosis and treatment. However, these results are consistent with previous work suggesting that symptoms are dominant in decisions to proceed to hospital [23]. The results also confirm anthropological work [39], carried out in Burkina Faso which suggests that treatment for symptoms corresponding with the biomedical picture of cerebral malaria and characterized by convulsions and coma *(kono*, a “bird-illness”) is the domain of *guérisseurs* (traditional health practitioners), used first, and often because traditional treatment is cheaper than the formal healthcare services; modern care occurs later. In contrast, the word *sumaya* linked to symptoms of fever, weakness, cold, loss of appetite, general body pain, diarrhoea, and vomiting is usually treated at home, by mothers, with herbs or paracetamol. It is, therefore, possible that parents considered that repeated vomiting and lethargy could be self-managed and delayed consultation with a modern provider; the delay was apparent even for those who eventually went to a health centre.

Household costs for children who sought care at health centre are higher than reported in other countries. In Uganda, the mean total out-of-pocket costs of patients who completed referral, some of whom received RA, were $2.52 [28] vs. $5.68 in Burkina Faso although only the costs after referral were calculated in Uganda. Since the full costs of the episode were calculated in this study, these data are unique in providing direct total household costs for an episode of severe illness, excluding the opportunity cost of time, for patients treated with RA in their community.

The non-RA children who went to a health centre paid on average $6.56 ($8.09 if they had CNS symptoms and $5.72 if they had repeated vomiting/lethargy). Menon et al. [24] found similar results ($6.84) in Uganda and other studies reported similar out-of-pocket costs (Ghana: $6.40 for severe cases; Malawi: $5.30 for children under 5) [25, 26].

The analysis has been limited to financial costs incurred by patients who survived the episode until the interview. The opportunity cost of labour, either forfeited to look after a sick child or withdrawn from agriculture because of the episode, was not calculated. It was not possible to obtain costs of RA-treated patients who survived the episode but died before the interview date because this would have been culturally insensitive. RDT results were not available for children in the non-RA group, and many of these children may not have had malaria, whereas all children in the RA-treated group had RDT-confirmed malaria. A further limitation is that, although all cost and time data relied upon memory, the data for the non-RA treated patients was obtained within 2 weeks of the episode, whereas data for the RA-treated patients were collected several months after the episode. It is difficult to determine whether costs in the RA-treated group were over or under-reported.

For several children in each group, the parents could not recall the exact date and/or time of symptoms/visits to a provider, and this resulted in incomplete information on time-intervals for several patients. Deterioration to CNS symptoms between visiting the last treatment provider and arrival at the health centre was used as an exploratory analysis of the higher drugs costs of the non-RA group. Although it is possible that higher drug costs could have been due to clinical deterioration, this hypothesis could not be confirmed. Furthermore, there was no attempt to ask qualitative questions about the reasons for not going to the health centre, and, for those who went, on the difficulties/barriers encountered in reaching the facility. With hindsight, such qualitative data would have been informative. Finally, it should be mentioned that RDTs and RA costs are not included in out-of-pocket costs as these were provided free of charge.

## Conclusions

This study reports evidence that children with CNS symptoms treated with RA incur similar household costs as children not treated with RA, despite the higher transport costs incurred in following the referral advice. Children without CNS symptoms treated with RA incurred higher total costs because of the costs of following referral advice. RA-treated children sought treatment more quickly, whether with or without CNS symptoms.

## Additional file

**Additional file 1.** Mean out-of-pocket household costs (US Dollars) for patients who incurred any costs for completed episodes of severe illness, by symptoms: children treated with RA versus children not treated with RA.

## Abbreviations

CHW: community health worker
CNS: central nervous system
CRF: case report form
CSPS: Centre de santé et de promotion sociale
iCCM: integrated community case management
IMCI: Integrated Management of Childhood Illness
RA: rectal artesunate
RDT: rapid diagnostic test

## References

[1] WHO, United Nations Children’s Fund (1999). Improving child health: IMCI: the integrated approach.

[2] Tulloch J (1999). Integrated approach to child health in developing countries. Lancet.

[3] Gomes MF, Faiz MA, Gyapong JO, Warsame M, Agbenyega T, Babiker A (2009). Pre-referral rectal artesunate to prevent death and disability in severe malaria: a placebo-controlled trial. Lancet.

[4] WHO, United Nations Children’s Fund (2005). Handbook IMCI: Integrated Management of Childhood Illness.

[5] WHO (2011). Integrated management of childhood illness: caring for newborns and children in the community.

[6] World Health Organization, United Nations Children’s Fund (2012). WHO/UNICEF Joint Statement Integrated Community Case Management (iCCM): an equity-focused strategy to improve access to essential treatment services for children.

[7] Bhutta ZA, Darmstadt GL, Hasan BS, Haws RA (2005). Community-based interventions for improving perinatal and neonatal health outcomes in developing countries: a review of the evidence. Pediatrics.

[8] WHO (2015). Guidelines for the treatment of malaria.

[9] Sauerborn R, Nougtara A, Diesfeld HJ (1989). Low utilisation of community health workers: results from a household interview survey in Burkina Faso. Soc Sci Med.

[10] Akweongo P, Agyei-Baffour P, Sudhakar M, Simwaka BN, Konaté AT, Adongo PB (2011). Feasibility and acceptability of ACT for the community case management of malaria in urban settings in five African sites. Malar J..

[11] Mukanga D, Tibenderana JK, Peterson S, Pariyo GW, Kiguli J, Waiswa P (2012). Access, acceptability and utilization of community health workers using diagnostics for case management of fever in Ugandan children: a cross-sectional study. Malar J..

[12] Druetz T, Kadio K, Haddad S, Kouanda S, Ridde V (2015). Do community health workers perceive mechanisms associated with the success of community case management of malaria? A qualitative study from Burkina Faso. Soc Sci Med.

[13] Druetz T, Ridde V, Kouanda S, Ly A, Diabaté S, Haddad S (2015). Utilization of community health workers for malaria treatment: results from a three-year panel study in the districts of Kaya and Zorgho, Burkina Faso. Malar J..

[14] Castellani J, Nsungwa-Sabiiti J, Mihaylova B, Ajayi IO, Siribié M, Afonne C (2016). Impact of improving community-based access to malaria diagnosis and treatment on household costs. Clin Infect Dis.

[15] Alemayehu T, Ghebreyesus TA, Witten KH, Bosman A, Teklehaimanot A (1998). Community-based malaria control programme in Tigray Region, Northern Ethiopia: results of a mortality survey of rural under-five children. Ethiop J Health Dev..

[16] Källander K, Hildenwall H, Waiswa P, Galiwango E, Peterson S, Pariyo G (2008). Delayed care seeking for fatal pneumonia in children aged under five years in Uganda: a case-series study. Bull World Health Organ.

[17] de Savigny D, Mayombana C, Mwageni E, Masanja H, Minhaj A, Mkilindi Y (2004). Care-seeking patterns for fatal malaria in Tanzania. Malar J..

[18] Müller O, Traoré C, Becher H, Kouyaté B (2003). Malaria morbidity, treatment-seeking behaviour, and mortality in a cohort of young children in rural Burkina Faso. Trop Med Int Health..

[19] Hill Z, Kendall C, Arthur P, Kirkwood B, Adjei E (2003). Recognizing childhood illnesses and their traditional explanations: exploring options for care-seeking interventions in the context of IMCI strategy in rural Ghana. Trop Med Int Health..

[20] Sicuri E, Vieta A, Lindner L, Constenla D, Sauboin C (2013). The economic costs of malaria in children in three sub-Saharan countries: Ghana, Tanzania and Kenya. Malar J..

[21] Chima RI, Goodman CA, Mills A (2003). The economic impact of malaria in Africa: a critical review of the evidence. Health Policy.

[22] Källander K, Tomson G, Nsungwa-Sabiiti J, Senyonjo Y, Pariyo G, Peterson S (2006). Community referral in home management of malaria in western Uganda: a case series study. BMC Int Health Hum Rights..

[23] Castellani J, Mihaylova B, Evers SM, Paulus AT, Mrango ZE, Kimbute O (2015). Out-of-pocket costs and other determinants of access to healthcare for children with febrile illnesses: a case-control study in rural Tanzania. PLoS ONE.

[24] Menon MP, Njau JD, McFarland DA (2016). Cost and predictors of care-seeking behaviors among caregivers of febrile children—Uganda 2009. Am J Trop Med Hyg.

[25] Asenso-Okyere WK, Dzator JA (1997). Household cost of seeking malaria care. A retrospective study of two districts in Ghana. Soc Sci Med..

[26] Hennessee I, Chinkhumba J, Briggs-Hagen M, Bauleni A, Shah MP, Chalira A (2017). Household costs among patients hospitalized with malaria: evidence from a national survey in Malawi, 2012. Malar J..

[27] Tozan Y, Klein EY, Darley S, Panicker R, Laxminarayan R, Breman JG (2010). Prereferral rectal artesunate for treatment of severe childhood malaria: a cost-effective analysis. Lancet.

[28] Nanyonjo A, Bagorogoza B, Kasteng F, Ayebale G, Makumbi F, Tomson G (2015). Estimating the cost of referral and willingness to pay for referral to higher-level health facilities: a case series study from an integrated community case management programme in Uganda. BMC Health Services Res.

[29] Simba DO, Warsame M, Kimbute O, Kakoko D, Petzold M, Tomson G (2009). Factors influencing adherence to referral advice following pre-referral treatment with artesunate suppositories in children in rural Tanzania. Trop Med Int Health.

[30] Ajayi IO, Nsungwa-Sabiiti J, Siribié M, Falade CO, Sermé L, Balyeku A (2016). Feasibility of diagnosis and management of malaria in Burkina Faso, Nigeria & Uganda: a community based observational study. Clin Infect Dis.

[31] Siribié M, Ajayi IO, Nsungwa-Sabiiti J, Afonne C, Balyeku A, Falade CO (2016). Training community health workers to manage uncomplicated and severe malaria: experience from 3 rural malaria-endemic areas in Sub-Saharan Africa. Clin Infect Dis.

[32] World Health Organization (2014). Severe malaria. Trop Med Int Health..

[33] Molyneux ME (1989). Malaria–clinical features in children. J R Soc Med.

[34] Von Seidlein L, Olaosebikan R, Hendriksen IC, Lee SJ, Adedoyin OT, Agbenyega T (2012). Predicting the clinical outcome of severe falciparum malaria in african children: findings from a large randomized trial. Clin Infect Dis.

[35] Phiri TB, Kaunda-Khangamwa BN, Bauleni A, Chimuna T, Melody D, Kalengamaliro H (2016). Feasibility, acceptability and impact of integrating malaria rapid diagnostic tests and pre-referral rectal artesunate into the integrated community case management programme A pilot study in Mchinji district, Malawi. Malar J.

[36] Siribié M, Ajayi IO, Nsungwa-Sabiiti J, Sanou AK, Jegede AS, Afonne C (2016). Compliance with referral advice after treatment with prereferral rectal artesunate: a study in 3 Sub-Saharan African countries. Clin Infect Dis.

[37] Singlovic J, Ajayi IO, Nsungwa-Sabiiti J, Siribié M, Sanou AK, Jegede AS (2016). Compliance with malaria rapid diagnosis testing by community health workers in 3 malaria-endemic countries of Sub-Saharan Africa: an observational study. Clin Infect Dis.

[38] Hains IM, Marks A, Georgiou A, Westbrook JI (2011). Non-emergency patient transport: what are the quality and safety issues? A systematic review. Int J Qual Health Care.

[39] Beiersmann C, Sanou A, Wladarsch E, De Allegri M, Kouyaté B, Müller O (2007). Malaria in rural Burkina Faso: local illness concepts, patterns of traditional treatment and influence on health-seeking behaviour. Malar J..

