# Supplementary material for: Household costs and time to treatment for children with severe febrile illness in rural Burkina Faso: the role of rectal artesunate
Source: Malar J. 2018 Oct 22;17:380. doi: 10.1186/s12936-018-2526-8 (PMC6198525; doi:10.1186/s12936-018-2526-8)
Supplement: Supplementary file 1 — Additional file 1. Mean out-of-pocket household costs (US Dollars) for patients who incurred any costs for completed episodes of severe illness, by symptoms: children treated with RA versus children not treated with RA. [file 12936_2018_2526_MOESM1_ESM.doc]

**Additional Table S1. Mean out-of-pocket household costs (US Dollars) for patients who incurred any costs for completed episodes of severe illness, by symptoms: children treated with RA versus children not treated with RA**

|  | **CNS + other symptoms** † | | | | | | **No CNS but prostrated with other symptoms** † | | | | | | **Total** | | | | | |
| --- | --- | --- | --- | --- | --- | --- | --- | --- | --- | --- | --- | --- | --- | --- | --- | --- | --- | --- |
|  | **RA** | | **No RA** | | **No RA but went to a health centre** | | **RA** | | **No RA** | | **No RA but went to a health centre** | | **RA** | | **No RA** | | **No RA but went to a health centre** | |
| **Category** | **N** | **Mean (SD)** | **N** | **Mean (SD)** | **N** | **Mean (SD)** | **N** | **Mean (SD)** | **N** | **Mean (SD)** | **N** | **Mean (SD)** | **N** | **Mean (SD)** | **N** | **Mean (SD)** | **N** | **Mean (SD)** |
| **Costs in USD** |  |  |  |  |  |  |  |  |  |  |  |  |  |  |  |  |  |  |
| **Registration** | - | - | - | - | - |  | 1 | 0.34 (-) | - | - | - | - | 1 | 0.34 (-) | - | - | - | - |
| **Consultation** | - | - | 11 | 0.32 (0.05) | 11 | 0.32 (0.05) | - | - | 13 | 0.33 (0.05) | 13 | 0.33 (0.05) | - | - | 24 | 0.32 (0.05) | 24 | 0.32 (0.05) |
| **Diagnosis** | 1 | 10.16 (-) | - | - | - | - | - | - | - | - | - | - | 1 | 10.16 (-) | - | - | - | - |
| **Drugs before health centre** | 10 | 0.16 (0.03) | 11 | 1.63 (3.04) | 2 | 0.17 (0.00) | 11 | 0.17 (0.00) | 21 | 0.46 (0.80) | 4 | 0.06 (0.07)* | 21 | 0.16 (0.02) | 32 | 0.86 (1.93) | 6 | 0.09 (0.08)** |
| **Drugs at the health centre** ‡ | 5 | 6.10 (8.00) | 9 | 6.03 (1.98) | 9 | 6.03 (1.98) | 15 | 6.19 (3.44) | 15 | 5.39 (3.90) | 15 | 5.39 (3.90) | 20 | 6.17 (4.72) | 24 | 5.63 (3.28) | 24 | 5.63 (3.28) |
| **Bed** | 1 | 3.39 (-) | 3 | 0.96 (0.20) | 3 | 0.96 (0.20) | 1 | 3.22 (-) | - | - | - | - | 2 | 3.30 (0.12) | 3 | 0.96 (0.20)* | 3 | 0.96 (0.20)* |
| **Food** ¶ | 25 | 1.88 (1.83) | 8 | 1.40 (1.42) | 7 | 1.52 (1.48) | 53 | 2.37 (2.53) | 6 | 0.40 (0.35) | 6 | 0.40 (0.35) | 78 | 2.21 (2.33) | 14 | 0.97 (1.18) | 13 | 1.01 (1.22) |
| **Other** | - | - | - | - | - | - | - | - | 1 | 0.51 (-) | 1 | 0.51 (-) | - |  | 1 | 0.51 (-) | 1 | 0.51 (-) |
| **Transport** | 30 | 2.74 (1.05) | 12 | 1.81 (0.80)* | 9 | 1.92 (0.76)** | 69 | 2.38 (1.00) | 19 | 1.41 (0.54)* | 18 | 1.45 (0.53)* | 99 | 2.49 (1.03) | 31 | 1.57 (0.67)* | 27 | 1.61 (0.64)* |
| **Total costs** | 30 | 5.83 (7.82) | 20 | 5.58 (4.73) | 11 | 8.09 (3.96) | 69 | 5.62 (4.18) | 37 | 3.36 (4.14)** | 20 | 5.72 (4.37) | 99 | 5.68 (5.50) | 57 | 4.14 (4.44) | 31 | 6.56 (4.32) |
| **Households that incurred cost** | 100% | - | 83% | - | 100% | - | 100% | - | 77% | - | 100% | - | 100% | - | 79% | - | 100% | **-** |

*RA* rectal artesunate, *CNS* central nervous system (convulsions; altered consciousness/coma), *SD* Standard deviation, *USD* US Dollars

* Reference group: RA – p<0.001

** Reference group: RA – p<0.05

† Repeated vomiting +/- too weak to take oral medication/“lethargy”

‡ For the non-RA group, only those who went to a health centre were included. Therefore, the results are the same as for the non-RA group who went to a health centre

¶ Food for the patient and accompanying family members
